# Assessing How the Aluminum-Resistance Traits in Wheat and Rye Transfer to Hexaploid and Octoploid Triticale

**DOI:** 10.3389/fpls.2018.01334

**Published:** 2018-10-15

**Authors:** Peter R. Ryan, Dengfeng Dong, Felix Teuber, Neele Wendler, Karl H. Mühling, Jie Liu, Muyun Xu, Naike Salvador Moreno, Jiangfeng You, Hans-Peter Maurer, Walter J. Horst, Emmanuel Delhaize

**Affiliations:** ^1^CSIRO Agriculture and Food, Canberra, ACT, Australia; ^2^College of Agriculture, Guangxi University, Nanning, China; ^3^Institute of Plant Nutrition and Soil Science, Kiel University, Kiel, Germany; ^4^College of Agronomy, Sichuan Agricultural University, Chengdu, China; ^5^Department of Genetics, Faculty of Biology, Universidad Complutense, Madrid, Spain; ^6^Laboratory of Soil and Plant Molecular Genetics, College of Plant Science, Jilin University, Changchun, China; ^7^State Plant Breeding Institute, Universitaet Hohenheim, Stuttgart, Germany; ^8^Institute for Plant Nutrition, Leibniz University Hanover, Hanover, Germany

**Keywords:** roots, acid soil, malate, citrate, *Secale cereal*e, *Triticum aestivum*

## Abstract

The mechanisms of aluminum (Al) resistance in wheat and rye involve the release of citrate and malate anions from the root apices. Many of the genes controlling these processes have been identified and their responses to Al treatment described in detail. This study investigated how the major Al resistance traits of wheat and rye are transferred to triticale (x *Tritosecale* Wittmack) which is a hybrid between wheat and rye. We generated octoploid and hexaploid triticale lines and compared them with the parental lines for their relative resistance to Al, organic anion efflux and expression of some of the genes encoding the transporters involved. We report that the strong Al resistance of rye was incompletely transferred to octoploid and hexaploid triticale. The wheat and rye parents contributed to the Al-resistance of octoploid triticale but the phenotypes were not additive. The Al resistance genes of hexaploid wheat, *TaALMT1*, and *TaMATE1B*, were more successfully expressed in octoploid triticale than the Al resistance genes in rye tested, *ScALMT1* and *ScFRDL2*. This study demonstrates that an important stress-tolerance trait derived from hexaploid wheat was expressed in octoploid triticale. Since most commercial triticale lines are largely hexaploid types it would be beneficial to develop techniques to generate genetically-stable octoploid triticale material. This would enable other useful traits that are present in hexaploid but not tetraploid wheat, to be transferred to triticale.

## Introduction

Many important crop species are stable allopolyploids resulting from hybridisations between two separate but related species. Triticale (× Triticosecale Wittmack) is an allopolyploid because it is a hybrid between rye (*Secale cereale* L.) and wheat (*Triticum aestivum* L.). Triticale is a valuable grain crop that combines useful traits from wheat and rye. Wheat has greater yield and superior grain quality while rye is a forage crop with outstanding resistance to many biotic and abiotic stresses including pathogens, low nutrient availability, soil pH and low temperatures. Wheat is typically used as the female parent and rye as the male parent because crosses are more stable if female plants have the larger ploidy of the two parents. The grain from this hybridisation are often sterile so the zygote from this cross is treated with colchicine to induce polyploidy and improve fertility (Mergoum and Gómez-Macpherson, 2004). When rye (diploid with genome RR) is hybridized with a hexaploid or bread wheat (hexaploid with genome AABBDD) the result is an “octoploid” triticale (AABBDDRR). When rye is hybridized with a tetraploid wheat (AABB) the result is a “hexaploid” triticale (AABBRR). Therefore triticale is amphidiploid meaning that it is diploid for the two parental genomes. Commercial triticale lines are mostly second generation hexaploid types because they often show better stability and performance than the octoploid types (Mergoum and Gómez-Macpherson, 2004).

Aluminum (Al) toxicity is a major limitation to crop production on acid soils because the concentration of soluble trivalent cations (Al^3+^) increases when soil pH falls below ~4.5. Many species show a significant genotypic variation in resistance to Al stress and this is also the case for rye and bread wheat but not for durum wheat which is very sensitive of Al. Rye is among the most Al-resistant cereal species along with rice (*Oryza sativa* L.). Aniol and Gustafson (1984) investigated the Al resistance of triticale, wheat and rye and concluded that Al resistance of the wheat parent was an important determinant of the Al resistance of triticale. They also found that the strong resistance of rye was partially suppressed in the hybrid. When that report was published little information was available on the mechanisms of Al resistance in any plant species. It was later revealed that the major mechanisms for Al resistance in wheat and rye involve the release or efflux of malate and citrate anions from the root apices (Li et al., 2000; Ma et al., 2000; Delhaize et al., 2007; Stass et al., 2008; Ryan et al., 2011). Stass et al. (2008) compared contrasting genotypes of wheat and rye with the triticale hybrids and concluded that the Al resistance of triticale was mostly determined by citrate efflux, a trait that was largely controlled by the wheat genome.

Differences in malate efflux account for most of the genotypic variation in Al resistance in bread wheat but citrate efflux is important when malate efflux is absent (Ryan et al., 2009). Malate efflux is facilitated by an anion channel encoded by the *aluminum-activated malate transporter, TaALMT1*, gene on chromosome 4DL (Sasaki et al., 2004; Raman et al., 2005). Al-resistant genotypes show a greater constitutive expression of *TaALMT1* in the root apices than sensitive genotypes which is not affected by Al treatment. However, the TaALMT1 protein requires Al^3+^ cations to trigger the malate release which means Al rapidly activates malate release (Sasaki et al., 2004). This rapid activation has been described as a Type I response which is consistent with the channel proteins being constitutively expressed and activated by Al (Ma et al., 2001). Citrate release from bread wheat is controlled by TaMATE1B, a transporter from the *multidrug and toxic compound exudation* (MATE) family (Ryan et al., 2009; Tovkach et al., 2013). TaMATE1B is encoded by a gene on chromosome 4BL. The greater citrate efflux is caused by a transposable element-like insertion near the transcription start site of *TaMATE1B* which results in a greater level of constitutive expression (Tovkach et al., 2013).

Members of these two gene families also control the release of malate and citrate from rye. Fontecha et al. (2007) identified a rye homolog of the wheat *TaALMT1* gene on chromosome 7RS and showed that its expression was induced by Al to a greater degree in the resistant cultivar *Ailés* than the sensitive cultivar *Riodeva*. Quantitative trait loci (QTL) for Al resistance were subsequently linked to this same region in two separate rye populations (Benito et al., 2005; Matos et al., 2005; Collins et al., 2008; Silva-Navas et al., 2012). In one of these populations, generated from the *M39A-1-6* (resistant) and *M77A-1* (sensitive) haplotypes, a cluster of *ScALMT* genes and one *ScMATE* gene was located on the 7RS locus (Collins et al., 2008). The resistant parent had five copies of the *ScALMT* gene and expression of two of these (*ScALMT1-M39.1* and *ScALMT1-M39.2*) was induced by Al in the root apices. By contrast, the sensitive parent had two copies of the *ScALMT* gene but only one (*ScALMT1-M77.1*) was induced by Al (Collins et al., 2008). Collins et al. (2008) was able to segregate the *MATE* gene from the resistance locus indicating that it was not contributing to the variation in Al resistance of that population. Those authors concluded that the *ScALMT* genes on 7RS controlled the Al-dependent efflux of malate from rye.

The first *MATE* gene in rye associated with citrate efflux from roots was the *ferric reductase-like 2* gene (*ScFRDL2*) (Yokosho et al., 2010). The expression of *ScFRDL2* in the roots was induced 15-fold by 50 μM Al and closely coincided with the Al-dependent changes in citrate efflux. Another *MATE* gene identified in the same study, *ScFRDL1*, was considered unlikely to be involved in Al resistance because it was induced by iron deficiency and not by Al treatment (Yokosho et al., 2010). Silva-Navas et al. (2012) later examined the population generated from *Ailés* and *Riodeva* and mapped a *MATE* gene which they named *aluminum-activated citrate transporter 1* (*ScAACT1*) in the Al-resistance QTL on chromosome 7RS. The authors proposed that *ScAACT1, ScFRDL1*, and *ScMATE* are all the same gene but this conclusion remains uncertain. For example, unlike *ScAACT1*, expression of *ScFRDL1* was not induced by Al treatment according to Yokosho et al. (2010) and the Al resistance QTL excluded the *MATE* gene in the population described by Collins et al. (2008). Silva-Navas et al. (2012) argued that these inconsistencies could be explained partly by differences in the parental lines and partly by differences in the length of treatments and Al concentrations used. Whereas Yokosho et al. (2010) used 50 μM Al treatments over 12 h, Silva-Navas et al. (2012) used 300 μM Al treatment over 24 h. The relatedness of these *MATE* genes requires further clarification.

The aim of the present study was to examine how well the Al-resistance traits in the wheat and rye parental lines were transferred to the allopolyploid triticale. Two sets of diverse lines were used for this purpose. One set included octoploid triticale lines generated from an Al-resistant rye and hexaploid wheat. The second set included hexaploid triticale lines generated by crossing a durum line with rye. Measurements were made of relative Al resistance, anion efflux and expression of selected Al-resistance genes in the parental material and triticale lines.

## Materials and methods

### Genetic material

Two sets of germplasm were used in the experiments (Table 1). The first set included two wheat cultivars, *Carazhino* and *Egret*, an Al-resistant rye line, ***L185***, and two second generation octoploid triticale lines generated from these wheat and rye parents. The triticale lines are depicted as *Carazinho*x***L185*** and *Egret*x***L185***. *Carazinho* is a highly Al-resistant wheat cultivar from Brazil that shows the Al-activated malate efflux controlled by *TaALMT1* and the constitutive release of citrate controlled by *TaMATE1B. Egret* is Al-sensitive and shows little or no organic anion efflux with or without Al treatment. The second set of germplasm included a tetraploid (durum) wheat named *5020-30*, an Al-resistant rye, ***390***, and a closely-related but Al-sensitive rye, ***389***, and the two primary hexaploid triticale lines derived from crossing these parents designated as *5020-30*x***390*** and *5020-30*x***389***. The triticale lines were generated at the University of Hohenheim, Germany.

**Table 1:** Summary of germplasm used in this study.

**Genotypes**	**Description**	**Al resistance mechanisms^†^**	**Al-resistance genes^††^**	**References**
**GERMPLASM SET 1**				
*Carazinho*	Wheat (hexaploid, Al-res)	Malate efflux Citrate efflux	*TaALMT1* *TaMATE1B*	Sasaki et al., 2004; Tovkach et al., 2013
*Egret*	Wheat (hexaploid, Al-sens)			
*Carazinho* x ***L185***	Triticale (octoploid)			
*Egret* x ***L185***	Triticale (octoploid)			
***L185***	Rye (diploid, Al-res)	Malate efflux Citrate efflux	*ScALMT1* *ScFRDL1 ScFRDL2 ScMATE* *ScAACT1*	Fontecha et al., 2007; Collins et al., 2008; Yokosho et al., 2010; Silva-Navas et al., 2012
**GERMPLASM SET 2**				
*5020–30*	Wheat (tetraploid, Al-sens)			
*5020–30* x ***389***	Triticale (hexaploid)			
*5020–30* x ***390***	Triticale (hexaploid)			
***389***	Rye (diploid, Al-sens)			
***390***	Rye (diploid, Al-res)	Malate efflux Citrate efflux		As above

† Likely mechanism from previous work but not previously investigated in these rye and triticale lines.

†† *These include known Al-resistance genes and candidate resistance genes. Note that some of the genes listed for citrate efflux might represent the same gene*.

### Aluminum resistance

Seeds were germinated for 2 days on moist filter paper and then planted over 20 L of aerated nutrient solution on laboratory benches. To estimate relative root length (RRL) the length of the longest root was measured before and after 4 days growth in the same nutrient solution with different Al concentrations. Therefore RRL was calculated as (net root growth in Al treatment net root growth in control solution) × 100.

### Measurement of citrate and malate efflux

The measurement of organic anion efflux from intact seedlings followed the procedures described previously (Delhaize et al., 1993; Ryan et al., 1995; Wang et al., 2007). Briefly, seeds were surface sterilized with bleach and thoroughly rinsed in sterile water. In preliminary experiments the seedlings were grown in aerated 20 L tubs with nutrient solution (pH 4.4) or in sterile conical flasks with 20 mL of 0.2 mM CaCl_2_ (pH 4.3) on a rotary shaker and exudates collected from excised roots. The large volume of the tubs maintained the root relatively free of microbial contamination and so both growth methods gave similar exudate results. Only results from the tubs are presented here. The excised root segments (eight to twelve per replicate) were washed in small vials with 1 mL of control solution (0.2 mM CaCl_2_, pH 4.3) for 1 h on a platform shaker (60 rpm). The solutions were rinsed and replaced by 1 mL of treatment solution (control solution with or without 40 μM AlCl_3_) and returned to the shaker for 2 h. After 2 h collection the malate and citrate concentrations in each solution were estimated enzymatically as described by Ryan et al. (1995). Malate assays used 0.1 mL of each sample and citrate assay used the remaining 0.9 mL. For the citrate assays the solutions were dried on a rotary vacuum drier and resuspended in 80 μL of assay solution as described by Ryan et al. (2009). All chemicals were obtained from Sigma-Aldrich Pty. Ltd. (Castle Hill, Australia). The concentrations were corrected to obtain the original malate and citrate contents in each sample and efflux was standardized for the number of apices and time of collection. In other experiments half of the seedlings were pretreated with 30 μM AlCl_3_ for at least 24 h prior to measurements as described in the figure legends.

### Measurements of gene expression

RNA was extracted from the root apices with the RNeasy PlantMini Kit (Qiagen) after grinding tissues in liquid nitrogen. cDNA was synthesized with the SuperScript III First-Strand Synthesis System (Invitrogen) as recommended using 1 μg RNA of each extraction. Gene expression was determined by qRT-PCR using the SYBR Green Supermix (Bio-Rad) kit on a Bio-Rad CFX96 Real Time System. Data were analyzed with the Bio-Rad CFX Manager software. The primers selected for measuring the expression of the Al-resistance genes in wheat and rye were specific for those genes and did not hybridize with sequences in the other species. Primers for *TaALMT1* expression in bread wheat were (5′-3′) CGTGAAAGCAGCGGAAAGCC (fwd) and CCCTCGACTCACGGTACTAACA (rev). Primers for *TaMATE1B* expression in bread wheat were AGGGTGGTAGCAGTGACTTC (fwd) and GCGGCAATCACCTTCTTGTG (rev). Annealing temperatures during cycling were 67°C for *TaMATE1B* and 61.5°C for *TaALMT1*. The primers for measuring *ScALMT1* expression in rye were GCAAACAATACCGTGGTTGTG (fwd) and ATCCCTCGAGTTAAGGCACC (rev). These primers could amplify products from the expressed copies of *ScALMT1* in the resistant and sensitive haplotypes of rye (*ScALMT1-M39.1, ScALMT1-M39.2, ScALMT1-M77.1*) described by Collins et al. (2008). We measured expression of *ScFRDL2* which is one of the candidate Al-resistance genes in rye because its Al-induced expression in the root apices by 50 μM Al is closely correlated with the release of citrate from roots (Yokosho et al., 2010). Primers used for measuring *ScFRDL2* expression were GGCTGCATTCCAGATTTGCTTG (fwd) and AGAAGCCCCAAGATCAATCCG (rev). Annealing temperatures were 68°C for *ScFRDL2* and *ScALMT1*.

The reference genes are important in the expression analyses because of the genetic differences between wheat and rye. Therefore the two reference genes chosen have previously been shown to be relatively stable across members of the triticaeae (Paolacci et al., 2009; Giménez et al., 2011). These gene are glyceraldehyde-3-phosphate dehydrogenase (*GAPDH*; Ta30768, Genbank EF592180) with primers GTTGAGGGTTTGATGACCAC (fwd) TCAGACTCCTCCTTGATAGC (rev) and the cell division control protein (AAA-superfamily of ATPases) (*CDC*; Ta54227) with primers GCCTGGTAGTCGCAGGAGAT (fwd) and ATGTCTGGCCTGTTGGTAGC (rev). In preliminary tests reliable amplicons were generated from wheat, rye and triticale with both sets of reference primers. Relative expression levels of the Al resistance genes were generally similar with both references genes and the results using *CDC* are presented.

### Statistical analysis

Al resistance was estimated by calculating relative root length (RRL) since this accounts for inherent differences in growth between different species (see above). Since RRL is a ratio of means (net root growth in different treatments) each of which has an error, then the result requires a new accumulated error. The formula for calculating this accumulated error and the procedure used for determining whether two RRL values are statistically different from one another is described previously by Zhou et al. (2013). The assumptions for this test are that the data are normally distributed and the variances are not different.

Other statistical analysis used the statistical software in SigmaPlot^TM^ ver 14.0. Anion efflux results were analyzed with a one way ANOVA. In cases where the data failed an initial normality test the data were first transformed with the natural log function (*ln*). Analyses were then determined by applying the Student-Newman-Keuls method for multiple pairwise comparisons. Analysis of gene expression was similar and used three biological replicates except as stated. Note that rye was not included in the analysis of the expression of wheat genes, and conversely, wheat lines were not included in the analysis of the expression of rye genes.

## Results

Two sets of germplasm were compared for Al resistance, organic anion efflux and expression of selected Al-resistance genes. The first set was comprised of two bread wheat cultivars (*Carazinho* and *Egret*), a rye cultivar (***L185***) and the two octoploid triticale lines generated from crossing the rye to each of the wheat cultivars (*Carazinho*x***L185*** and *Egret*x***L185***). *Egret* is an Al-sensitive cultivar that shows little or no malate or citrate release. *Carazinho* is an Al-resistant wheat that has the Al-resistant alleles for *TaALMT1* and *TaMATE1B. Carazinho* has greater expression of these two genes than *Egret* and displays an Al-activated efflux of malate and a constitutive release of citrate from the root apices (Ryan et al., 2009). The second set of germplasm included a tetraploid (durum) wheat line (*5020-30*), two closely-related lines of rye with contrasting resistance to Al (***390*** resistant and ***389*** sensitive) and the two hexaploid triticale lines generated by crossing the durum wheat with each rye line (*5020-30*x***390*** and *5020-30*x***389***). The mechanisms of Al resistance in the ***390*** have not previously been investigated in detail.

### Octoploid triticale

**Figure 1:**
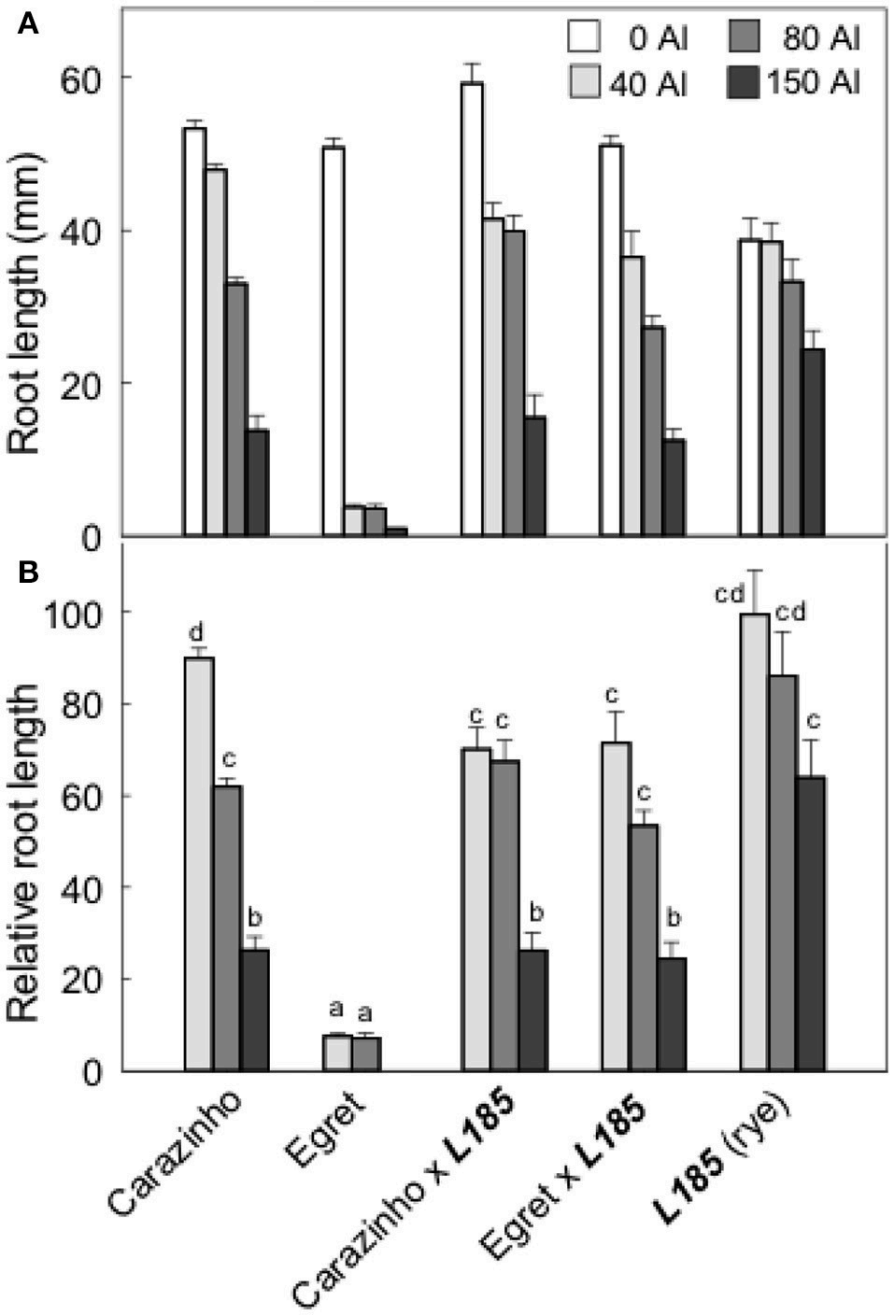
Al resistance of the wheat and rye parents and the resulting triticale lines. Root length after 4 days growth in a range of Al concentrations **(A)** and relative root length **(B)** was measured in two hexaploid wheats, *Carazinho* (Al-resistant) and *Egret* (Al-sensitve), a rye genotype ***L185***, and the triticales generated by crossing the rye with each wheat parent. Data show means, SE (*n* = 6–10). Data with different letters in **(B)** are significantly different from one another (*p* < 0.05).

Al resistance of the wheat, rye and octoploid triticale lines was compared by estimating relative root length after 4 d growth in a range of Al concentrations (Figure 1). Egret wheat was sensitive of all Al treatments while the Carazinho wheat and triticale lines were more resistant. At the highest Al treatment L185 rye was most resistant with 62% RRL while the wheat and triticale lines were similar at 25%. These results indicate that rye could contribute to the Al resistance of triticale because EgretxL185 triticale was significantly more resistant than Egret wheat. However, the Al resistance of rye and wheat was not additive in triticale because the resistance of CarazinhoxL185 was no greater than either the wheat or rye parent. It would be instructive to confirm the Al resistance measured in hydroponics reflects the measurements in field trials with acidic soil.

**Figure 2:**
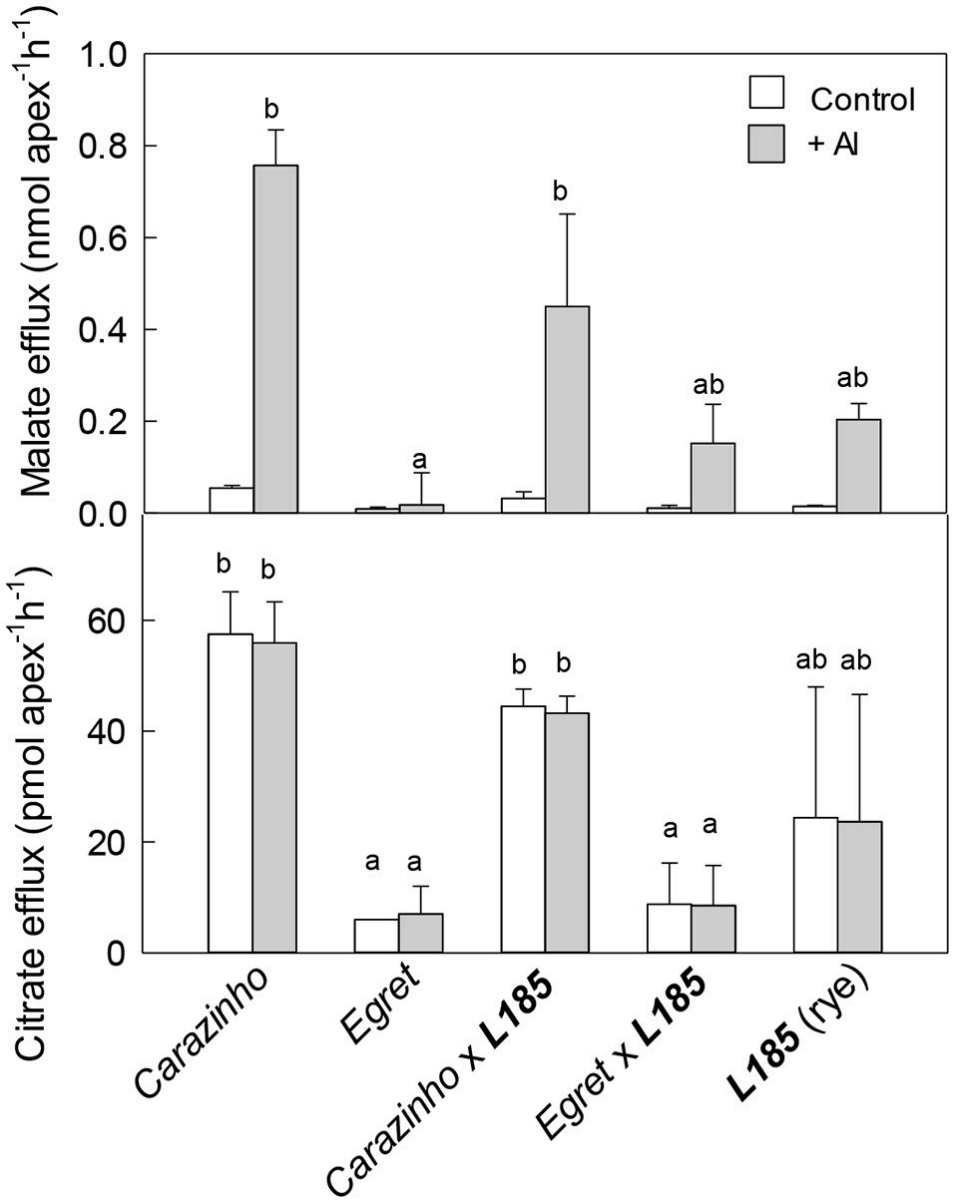
Malate and citrate efflux from genotypes in the presence and absence of Al. Malate and citrate efflux were measured from excised root apices over 2 h with and without 40 μM Al in the collection solution. Data show the mean and SE (*n* = 3 or 4). Data with different letters are significantly different from one another (*p* < 0.05). Note the statistical analysis in **(A)** included the +Al treatment only whereas in **(B)** both treatments were included.

Malate and citrate release are known mechanisms for Al resistance in rye and wheat and fluxes of these organic anions were measured from each genotype. The results showed some variation between replicated experiments, especially in the triticale lines and rye material so the experiments were repeated several times. In the first series of experiments seedlings were grown in control nutrient solution and then malate and citrate efflux were measured in the presence or absence of Al. This means that the root tips were only exposed to Al for 2 h as exudates were collected. In the absence of Al, malate efflux from all genotypes was less than 0.05 nmol apex^−1^ h^−1^ (Figure 2). When 40 μM Al was included in the treatment solution, malate efflux increased significantly in all genotypes except for *Egret* wheat. The largest malate release was from *Carazinho* and *Carazinho*x***L185***. This result is consistent with the Al-activation of malate efflux from wheat reported previously (Delhaize et al., 1993; Ryan et al., 1995) and indicates that the malate efflux trait from *Carazinho* wheat was fully expressed in the *Carazinho*x***L185*** triticale. Citrate efflux from *Carazinho* and *Carazinho*x***L185*** was large in the presence and absence of Al and indicates that citrate efflux was constitutive in these genotypes (Figure 2). Citrate efflux from *Egret* and *Egret*x***L185*** was smaller regardless of Al, while efflux from rye was very variable. These results support previous observations in *Carazinho* and *Egret* and indicate that the large constitutive efflux of citrate from *Carazinho* was also transferred to the triticale.

**Figure 3:**
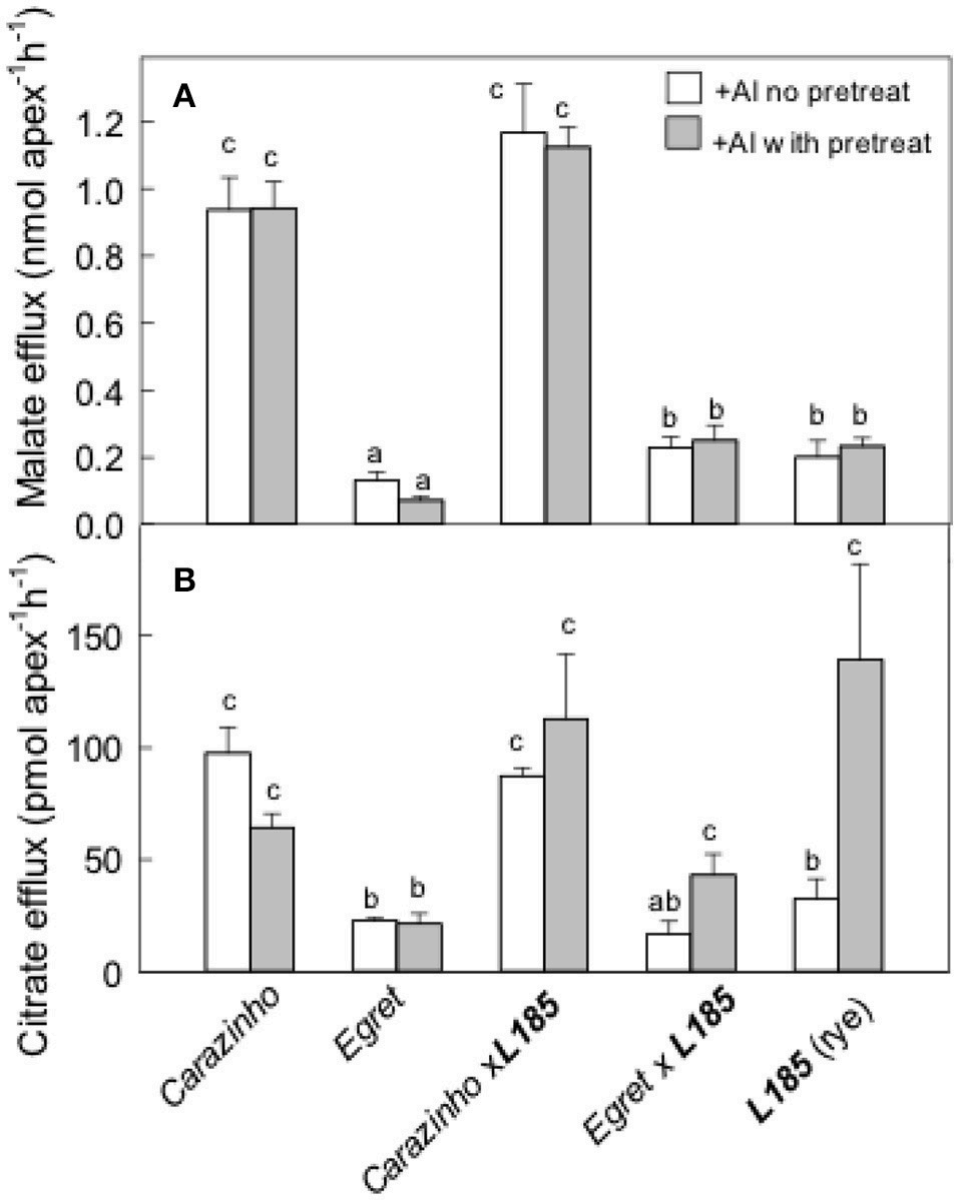
Malate and citrate efflux from genotypes with and without pre-treatment in Al. Malate **(A)** and citrate **(B)** efflux were measured from apices over 2 h in 40 μM Al with and without a pretreatment in 30 μM Al for at least 24 h. Mean ±SE (*n* = 3 or 4). Data with different letters indicate significant differences after a one factor ANOVA (*p* < 0.05) using the Student-Newman-Keuls method for multiple pairwise comparisons. The data in **(B)** were first transformed with natural logarithm to satisfy normality.

Organic anion efflux in some plant species is induced by Al treatment over many hours or longer (Pellet et al., 1995; Li et al., 2000; Ma et al., 2001; Magalhaes et al., 2007; Delhaize et al., 2012). Anion release was therefore measured after pretreating the seedlings in Al. In these experiments, half the seedlings were pretreated in 30 μM AlCl_3_ for at least 24 h prior to the measurements and the other seedlings were only exposed to Al during the 2 h collection period. The results in Figure 3A show that the pretreatment in Al did not affect malate efflux from any genotype. Efflux from *Carazinho* wheat and *Carazinho*x***L185*** triticale remained greater than from *Egret*, ***L185*** rye and *Egret*x***L185*** triticale. Citrate efflux from *Carazinho* and ***L185***x*Carazinho* was large and unaffected by pretreatment in Al (Figure 3B). Citrate efflux from ***L185*** rye increased significantly after Al pretreatment while efflux from ***L185***x*Egret* showed a small but significant increase following pretreatment. These results demonstrated the following: (i) the Al-activated efflux of malate and the constitutive efflux of citrate was fully transferred from *Carazinho* wheat to the *Carazinho*x***L185*** triticale; (ii) Al pretreatment enhanced the efflux of citrate but not of malate from ***L185*** rye; (iii) the citrate efflux phenotype in rye was not fully transferred to triticale.

**Figure 4:**
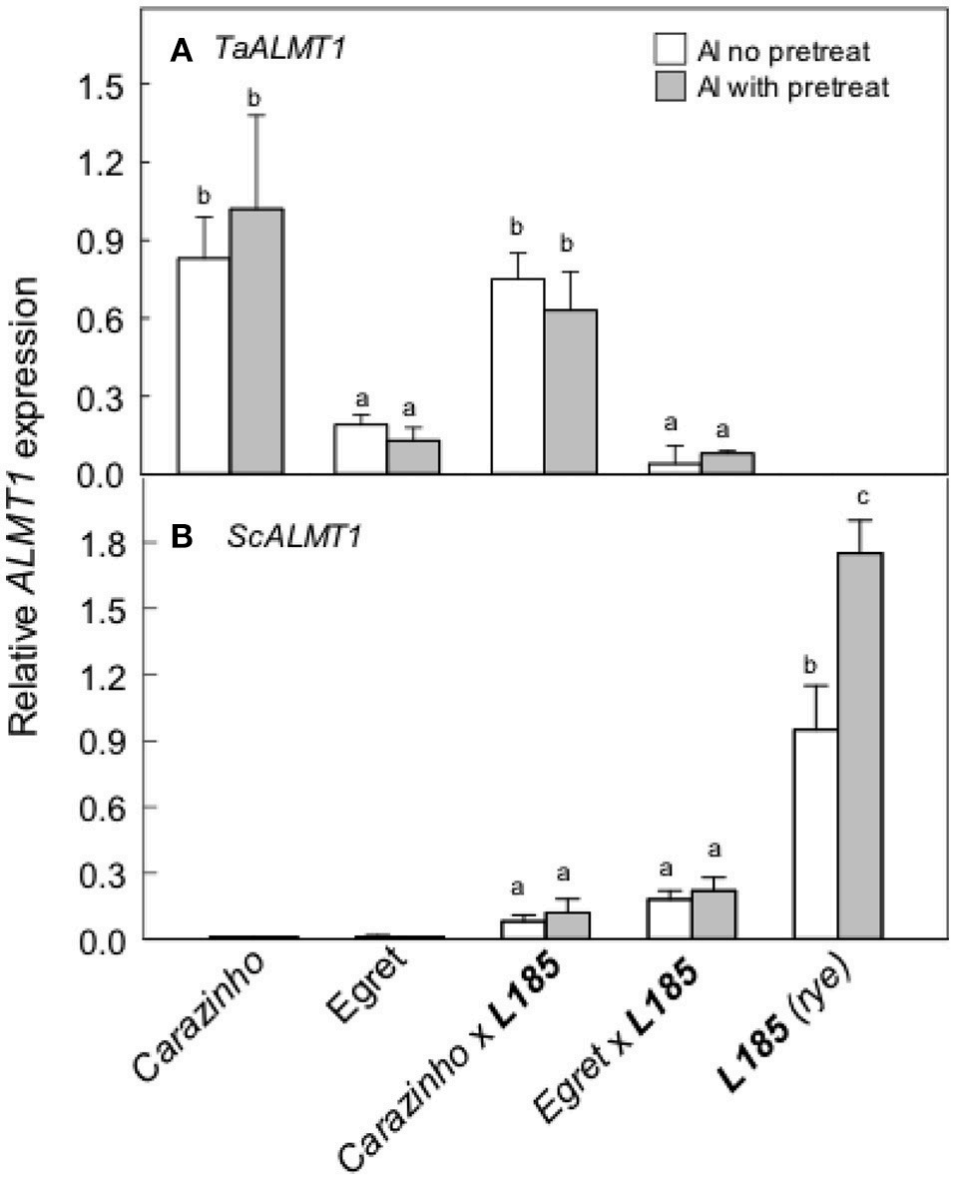
Expression of the *ALMT1*-type genes in wheat, rye and triticale lines. Relative expression of the *TaALMT1*** (A)** and the *ScALMT1*** (B)** genes was measured with or without a pretreatment in 30 μM AlCl_3_ for at least 24 h. Data show means and SE (*n* = 3 biological replicates). Data with different letters indicate significant differences at *p* < 0.05 using a one factor ANOVA.

We next measured the expression of the *ALMT* genes, *TaALMT1* and *ScALMT1*, that control malate release from wheat and rye roots. Note that the primers used for *ScALMT1* recognize several copies of the *ScALMT1* genes located in the *Alt4* locus in rye as reported by Collins et al. (2008). Expression of the wheat gene *TaALMT1* was greater in *Carazinho* and *Carazinho*x***L185*** than the other genotypes and unaffected by pretreatment with Al (Figure 4A). *TaALMT1* expression was low in *Egret* and *Egret*x***L185*** as expected. *ScALMT1* expression was significantly greater in ***L185*** rye than the two triticale lines (Figure 4B). Pretreatment tended to induce expression but the difference in this experiment was not significant. These results indicate that expression of the rye *ScALMT1* gene was suppressed in octoploid triticale.

**Figure 5:**
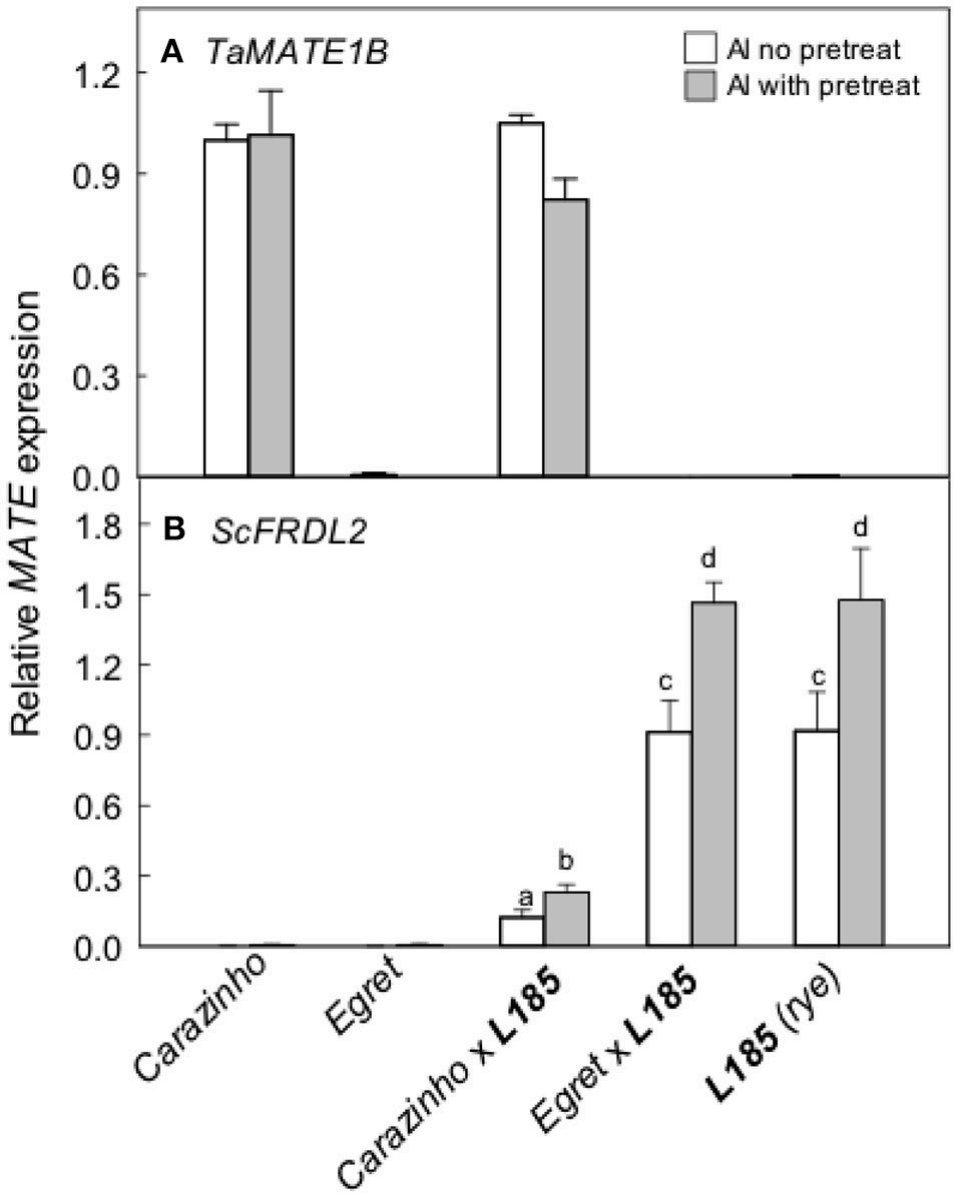
Expression of specific *MATE*-type genes in wheat, rye and the triticale lines. Relative expression of the *TaMATE1B*** (A)** and the *ScFRDL2*** (B)** genes was measured with and without a pretreatment in 30 μM AlCl_3_ for 24 h. Means, SE (*n* = 3 biological replicates for *TaMATE1B* and *n* = 3–4 for *ScFRDL2*). Data with different letters indicate significant differences after a one way ANOVA (*p* < 0.05) using the Student-Newman-Keuls method for multiple pairwise comparisons. The data in **(B)** were first transformed with natural logarithm to satisfy normality.

The *TaMATE1B* and *ScFRDL2* genes encode transporters that likely facilitate citrate efflux from wheat and rye respectively. *TaMATE1B* expression levels were high in *Carazinho* and *Carazinho*x***L185*** and unaffected by pretreatment with Al (Figure 5A). Little or no expression was detected in *Egret* and *Egret*x***L185***. These data indicate that *TaMATE1B* was expressed similarly in wheat and triticale. *ScFRDL2* expression was detected in ***L185***, *Egret*x***L185*** but it was suppressed in *Carazinho*x***L185*** (Figure 5B). These data indicate that the rye *ScFRDL2* gene was expressed in triticale but the level of expression varied with the different wheat parents.

### Hexaploid triticale

**Figure 6:**
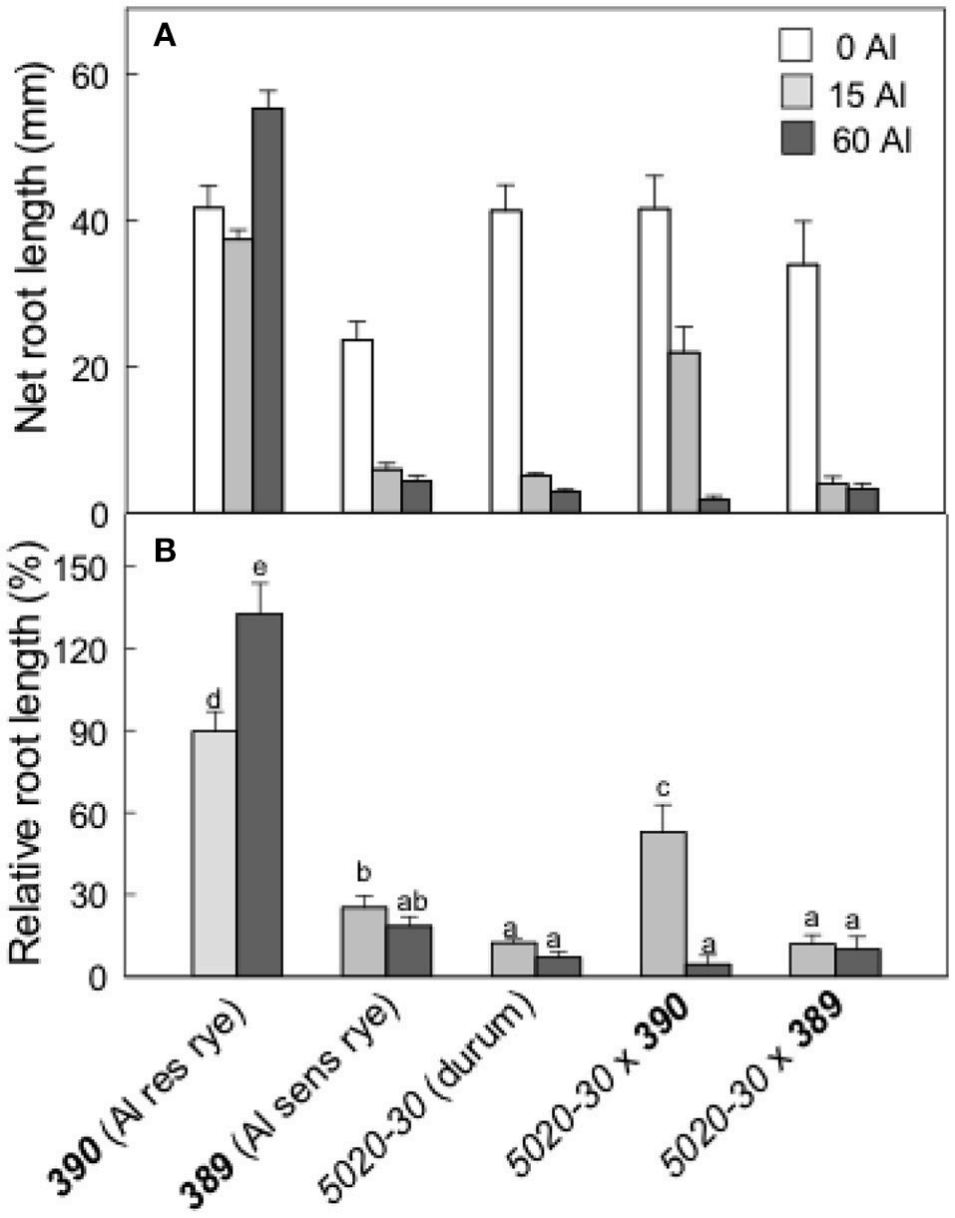
Al resistance of the wheat, rye and triticale lines. Net root length in a range of Al concentrations **(A)** after 4 days and relative root length **(B)** in Al-resistant (***390***) and sensitive (***389***) rye lines, a durum wheat (tetraploid) and the triticale lines. Data show means with SE (*n* = 6–10). Data with the different letters in **(B)** are significantly different at *p* < 0.05.

The second set of experiments examined the rye lines ***390*** and ***389***, a tetraploid (durum) wheat line (*5020-30*) and the two hexaploid triticale lines generated from crossing each rye line to the durum wheat (*5020-30*x***390*** and *5020-30*x***389***). The ***390*** rye is resistant to Al and ***389*** is closely related but more sensitive to Al. The Al resistance of these lines was compared by estimating relative root length after 4 d growth in 0, 15, and 60 μM Al (Figure 6). Rye ***390*** showed no inhibition of root growth at 60 μM Al which is consistent with it being the most resistant genotype. RRL for most other genotypes was 20% or less for all treatments. The exception was *5020-30*x***390*** triticale where RRL was ~50% in 15 μM Al (Figure 6B). These data indicate that the Al resistance of the ***390*** rye was incompletely transferred to hexaploid triticale.

**Figure 7:**
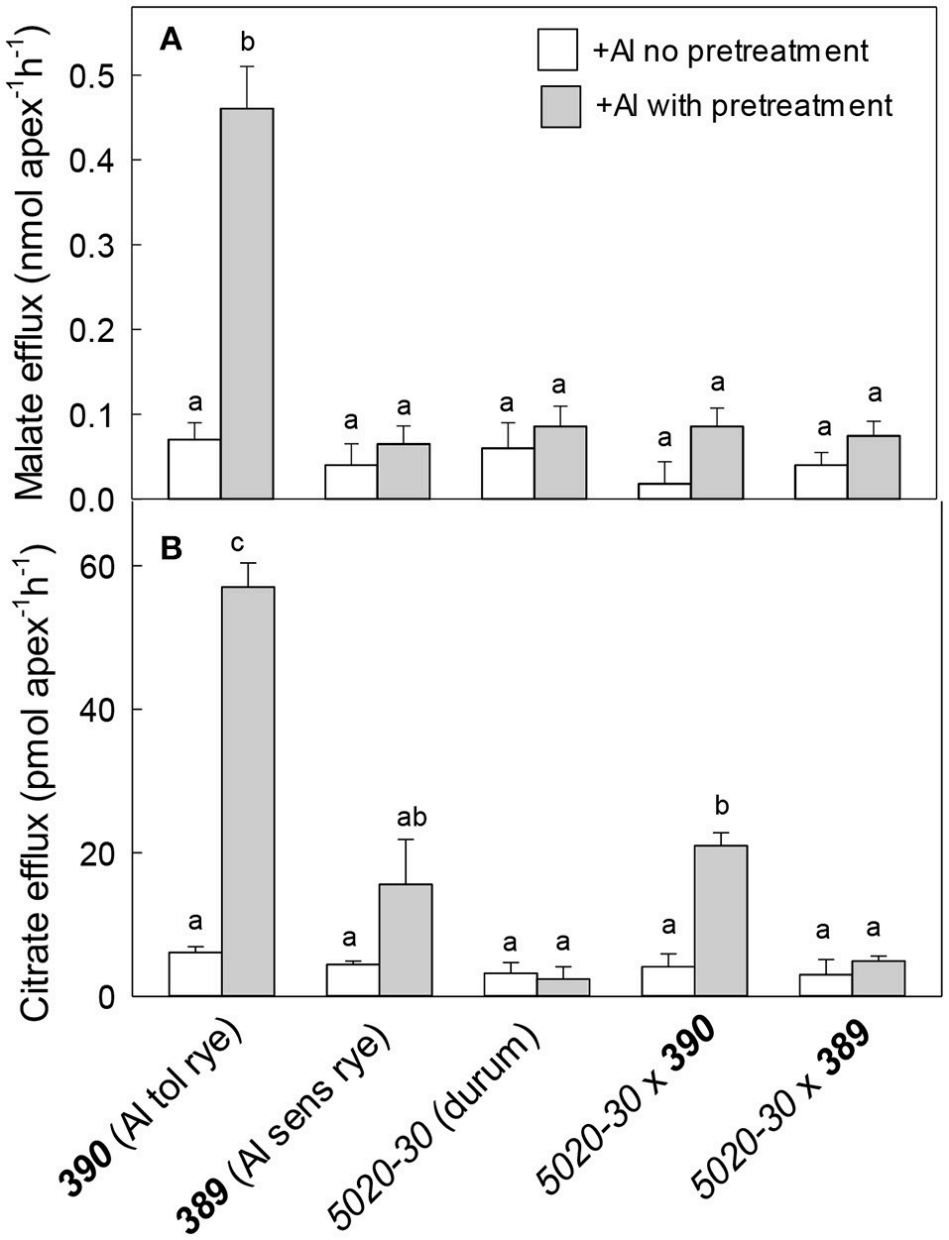
Malate and citrate efflux from wheat, rye and triticale with and without a pre-treatment in Al. Malate **(A)** and citrate **(B)** efflux were measured in the presence of 40 μM Al with and without a pre-treatment in 30 μM Al for at least 24 h. Data show mean and SE (*n* = 3 or 4). Data with different letters are significantly different at (*p* < 0.05) after a one factor ANOVA.

Malate and citrate efflux from these genotypes was measured with or without a pretreatment in 30 μM Al (Figure 7). Malate efflux from the Al-resistant rye ***390*** was induced by Al pretreatment and was five-fold greater than the other genotypes (Figure 7A). Citrate efflux from the resistant ***390*** rye showed a large induction by Al pretreatment while efflux from the *5020-30*x***390*** triticale showed a smaller induction reaching only ~30% of the rye (Figure 7B). Efflux from the other genotypes remained small. These results show that malate and citrate efflux likely contribute to the Al resistance of the ***390*** rye. They also indicate that the malate efflux detected in the ***390*** rye was not transferred to the hexaploid triticale *5020-30*x***390*** while citrate efflux was only partially transferred to triticale.

**Figure 8:**
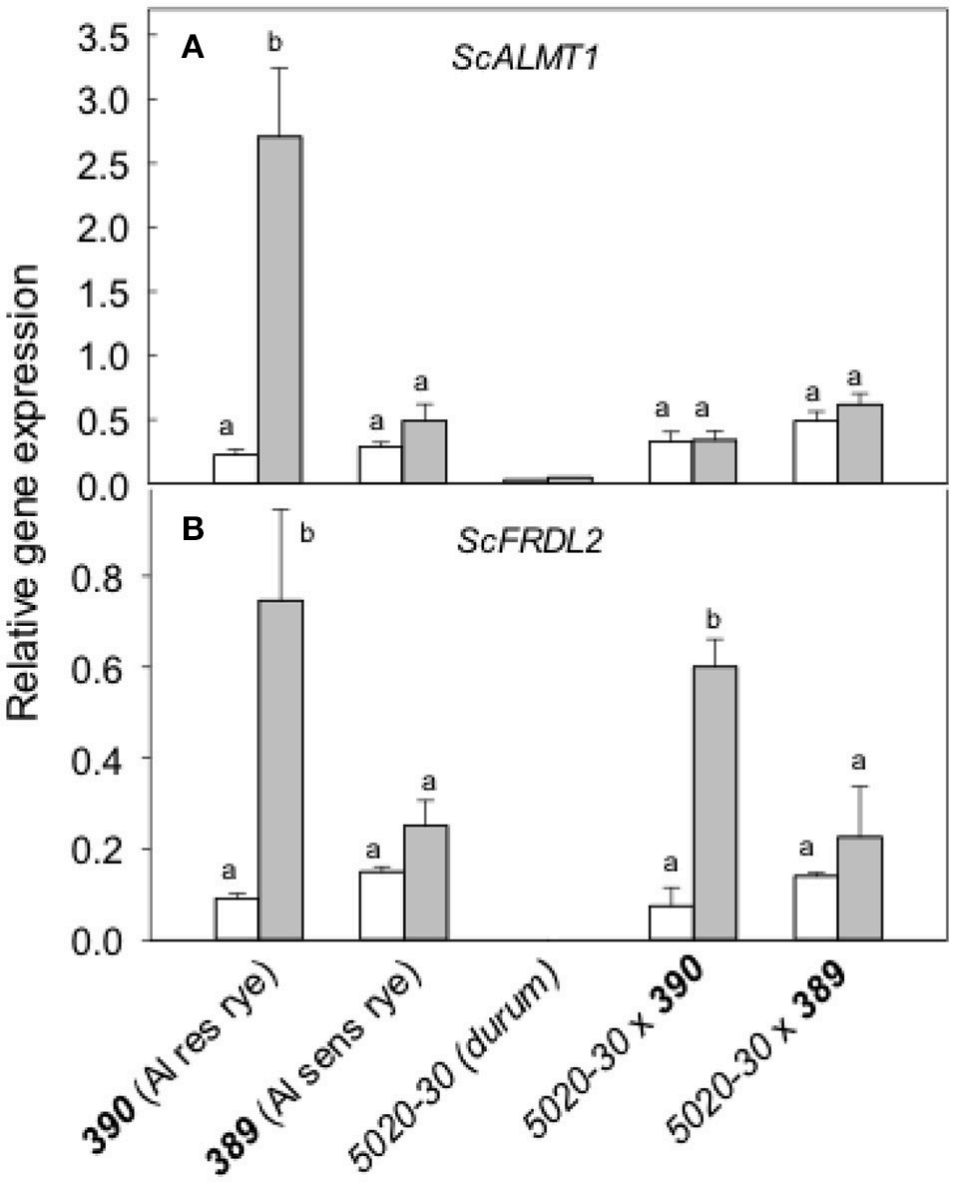
Relative expression of the rye *ScALMT* and *ScFRDL2* genes in different genotypes. Relative expression of *ScALMT*** (A)** and *ScFRDL2*** (B)** was measured without (white) and with (gray) a pre-treatment in 30 μM Al for at least 24 h. Data show mean and SE (*n* = 3 biological replicates). Data with different letters are significantly different at (*p* < 0.05) after a one factor ANOVA.

Expression of the rye genes contributing to malate and citrate, *ScALMT1* and *ScFRDL2* respectively, were then measured with and without pretreatment in Al (Figure 8A). Without a pretreatment, *ScALMT1* expression was low in all lines. After a pretreatment in Al, *ScALMT1* expression in ***390*** rye increased 10-fold but was not induced in any of the other lines. These responses are consistent with the measured efflux of malate. The expression of *ScFRDL2* was significantly increased by Al pretreatment in ***390*** rye and *5020-30*x***390*** triticale but remained lower in the other lines (Figure 8B). These results indicate that the *ScFRDL2* gene was induced by Al in the Al-resistant ***390*** rye and the *5020-30*x***390*** triticale but that expression of *ScALMT1* was suppressed in the *5020-30*x***390*** triticale.

## Discussion

Al-resistance in hexaploid wheat and rye relies on the efflux of malate and citrate anions from the root apices. These phenotypes are controlled in part by the *TaALMT1* and *TaMATE1B* genes in hexaploid wheat and by the *ScALMT* and *ScFRDL2* genes in rye. This study investigated the transfer of these resistance mechanisms from wheat and rye lines to triticale. The first set of germplasm examined included Al-sensitive (*Egret*) and resistant (*Carazinho*) hexaploid wheat cultivars, an Al-resistant rye (***L185***) and the two octoploid triticale lines generated by crossing each wheat with the rye. All of these lines except for the *Egret* wheat showed strong resistance to Al toxicity (Figure 1). The following conclusions can be made from these first set of lines: (1) The wheat and rye parents both contributed to the Al-resistance of octoploid triticale. Support for this conclusion comes from the finding that *Egret*x***L185*** triticale was significantly more Al-resistant than *Egret* (Figure 1) which indicates that ***L185*** contributed to the phenotype. Further, the malate and citrate efflux in *Carazinho*x***L185*** resembled the responses in *Carazinho* wheat but not that of rye (Figures 2, 3) indicating that wheat contributed to those traits. These responses generally reflected the relative expression of the genes involved. (2) Al resistance of the parental lines was not additive in triticale. This supported by the finding that neither triticale line was more resistant than the rye or Al-resistant wheat parents. (3) Function of the Al resistance genes in hexaploid wheat were more completely transferred to triticale than the rye genes. This is shown by the expression levels of the two wheat genes *TaALMT1* and *TaMATE1B* which were similar in *Carazinho* and *Carazinho*x***L185*** whereas expression of the rye genes, *ScALMT* and *ScFRDL2*, in triticale was inconsistent (Figures 4, 5). In a previous study, Stass et al. (2008) concluded that the Al resistance of triticale was determined by citrate efflux which was largely controlled by the wheat parent. The present results indicate that malate efflux from hexaploid wheat can also contribute to the resistance of triticale. (4) The expression of the rye *ScFRDL2* gene in triticale depended on the genotype of the wheat parent. This is supported by the observations that relative expression of *ScFRDL2* was significantly greater in *Egret*x***L185*** than *Carazinho*x***L185*** (Figure 5). It is interesting to speculate whether this finding is related to the different expression levels of the wheat gene *TaMATE1B* in *Carazinho* and *Egret*.

The second set of germplasm included a durum wheat (*5020-30*), a pair of closely-related rye lines (***389*** and ***390***) that differed in Al resistance and the two hexaploid triticale lines generated by crossing each rye with the durum. The main conclusions drawn from those results include the following: (1) Malate and citrate efflux contribute to the Al resistance of ***390*** rye and these fluxes were correlated with increases in *ScALMT1* and *ScFRDL2* expression in ***390*** rye. (2) The Al resistance of 390 rye was not fully transferred to the 5020-30 x 390 triticale (Figure 6). This was consistent with the reduced efflux of organic anions from 5020-30 x 390 compared to the resistant rye (Figure 8). The Al resistance of these lines appeared to be most closely correlated with citrate efflux and expression of ScFRDL2.

The important observation from both sets of lines was that the Al resistance of rye was incompletely transferred to triticale—whether to a hexaploid or a octoploid triticale. Similar observations have been made previously for other rye genes in triticale (Neves et al., 1995; Kalinka and Achrem, 2018). By contrast, the Al resistance traits from hexaploid wheat did transfer more successfully to octoploid triticale.

The incomplete transfer of the Al-resistance traits of rye to triticale may be explained by the modifications that commonly occur to the genome of *de novo* allopolyploids mentioned above. When related species such as wheat and rye hybridize to form a stable allopolyploid many genes become duplicated and genetic changes that occur can affect gene expression. Furthermore, homeolog copies of all genes in allopolyploids are not expressed equally. Sequences can be lost and mutations generated due to chromosomal rearrangements or transposon activity (Ma and Gustafson, 2008), and gene transcription can be affected by epigenetic modifications and microRNAs (Cheng and Murata, 2002; Kashkush et al., 2002, 2003; Kraitshstein et al., 2010; Li et al., 2014, 2015; Kalinka and Achrem, 2018). Cytosine residues in DNA are prone to methylation when they occur as CpG, CpHpG, and CpHpH sites (where H represents any nucleotide except guanine) and methylation of gene promoter regions can interfere with transcription. In *de novo* allopolyploids, such as the primary triticale lines used here, DNA methylation appears to be a more important factor decreasing gene expression than genetic instability with some estimates suggesting 1 to 12% of genes are silenced this way (Kashkush et al., 2002; He et al., 2003; Mochida et al., 2004; Bottley et al., 2006). The homeologous genes from one parent genome in *de novo* allopolyploids can be silenced more than the other parent and this can even vary between different organs (Bottley et al., 2006; Zhao et al., 2011).

We propose that the rye traits are incompletely transferred to triticale because its genome is naive to the polyploid environment and therefore more prone to epigenetic modification. Hexaploid wheat, by contrast, has emerged from two major hybridization events. The first hybridization occurred about 0.5 million years ago between a diploid species (likely *Triticum uratu, AA* genome) and another unknown parent with the *BB* genome which generated an ancestral tetraploid wheat. The second event occurred only 10,000 years ago between a tetraploid species such as *Triticum turgidum* (*BBAA*) and the diploid grass *Aegilops tauschii* (*DD*) and generated hexaploid wheat. Since wheat has been subject to epigenetic silencing pressure for a long period, its genome is likely to be more resistant to further silencing processes than the rye genome in a wheat-rye hybrid. This idea is consistent with the outcome of previous studies that compared the expression of genes in tetraploid and diploid lines with their expression in *de novo* hexaploid wheat lines. For example, microarray analysis and RNA-seq techniques demonstrated that more genes from the diploid parent had reduced expression levels in the hexaploid line than the tetraploid parent. This indicates an “expression bias” toward the tetraploid genome parent compared to the diploid genome (Akhunova et al., 2010; Li et al., 2014). Future work will test this hypothesis by investigating how methylation states of specific Al-resistance genes in the wheat and rye parents change in the primary triticale lines.

Significant variation was detected between certain repeated experiments and in some instances anion fluxes did not correlate well with gene expression. For example, the relative expression of *ScALMT1* in ***L185*** and *Egret*x***L185*** did not reflect the measured fluxes of malate. This variation could be related, in part, to the variable delay in gene induction by Al and the possible involvement of other Al resistance genes not targeted in this study. More than one *ALMT* and *MATE* gene could contribute to anion efflux (see Introduction). The variation may also be related to the instability of the primary triticale lines which can continue in subsequent generations (Ma and Gustafson, 2008; Kalinka and Achrem, 2018). The grain used in these experiments were bulked on two occasions so some variation was not unexpected. However each experiment was performed several times and the results presented here reflect the same general trends. Future studies could, nevertheless, quantify the stability of the triticale lines by determining the chromosome number of individual plants within each line and in different generations. The expression levels of target genes could also be measured and Al-resistance assessed in field trials on acidic and limed soils over several sites and seasons. These experiments would provide further insight into the genetic stability of the primary triticale material.

Triticale was developed to combine the favorable attributes of rye and wheat and to generate diversity. The triticale material used in this study were primary triticale lines generated by crossing rye pollen to female wheat plants. The reverse cross (rye as the female parent) is possible but less successful. Nevertheless, since the mitochondrial and plastid genomes are maternally inherited some traits are under cytoplasmic control (Thiede, 1998; Battich et al., 2015). Additional diversity might be generated if improved technologies enabled the reverse crosses to occur more efficiently so that the origins of the mitochondrial and plastid genomes in triticale would be rye instead of wheat.

Most triticale grown around the world are hexaploid types because they tend to show better vigor and stability (Mergoum et al., 2009). The present study found that the strong Al-resistance of rye was not fully expressed in either the hexaploid or octoploid triticale whereas the Al-resistance traits derived from Carazinho hexaploid wheat did transfer to octoploid triticale more successfully. If this pattern is indicative of other phenotypes then hexaploid triticale is potentially missing other valuable traits that may occur in some hexaploid wheat but not tetraploid wheat. Consideration should be given to developing cytological techniques that improve the genetic stability of octoploid triticale so that beneficial traits of hexaploid wheat (e.g., flour quality, nutrient content) can be captured in the hybrid. Such an approach could further improve the value of triticale production.

## Author contributions

WH and PR conceived the project and coordinated with the breeder to access the germplasm. Experimental word was performed by DD, NW, JL, MX, NSM, JY, ED and PR. Supervision of experimental work was provided by PR, ED and KM. The manuscript was drafted by PR with help from all authors.

### Conflict of interest statement

The authors declare that the research was conducted in the absence of any commercial or financial relationships that could be construed as a potential conflict of interest.
